# Incidence and predictors of immune checkpoint inhibitor treatment–related cognitive impairment in a racial and ethnic diverse population

**DOI:** 10.1007/s00520-025-09560-0

**Published:** 2025-06-02

**Authors:** Michael Sayer, Parisa Agrawal, Ding Quan Ng, Dalia Kagramanov, Julia Trudeau, Shivashankar Othy, Munjal M. Acharya, Alexandre Chan

**Affiliations:** 1 Department of Clinical Pharmacy Practice, School of Pharmacy & Pharmaceutical Sciences, University of California, 802 W Peltason Dr, Irvine, CA 92697-4625 USA; 2 Department of Physiology & Biophysics, School of Medicine, University of California, Irvine, CA USA; 3 Department of Anatomy & Neurobiology, School of Medicine, University of California, Irvine, CA USA; 4 Department of Radiation Oncology, School of Medicine, University of California, Irvine, CA USA

**Keywords:** Cancer-related cognitive impairment, Immune checkpoint inhibitors, Immunotherapy, Patient-reported outcomes

## Abstract

**Introduction:**

Immune checkpoint inhibitors (ICI) have revolutionized cancer therapy in recent years. In addition to rejuvenating anti-cancer immunity, ICI may cause immune dysregulation, impacting homeostasis, including brain functions. Thus, the association of ICI with cognitive function needs further investigation. Using NIH’s PROMIS system, this study investigates self-reported cognitive impairment within a diverse cohort of ICI-treated patients. Additionally, we explore risk factors influencing self-reported cognitive function, including concurrent symptoms and racial/ethnic background.

**Methods:**

This was a prospective, longitudinal study conducted between July 2021 and June 2023. Included patients were ≥ 18 years old, newly diagnosed with cancer, and scheduled to receive ICI therapy. Serial patient-reported outcomes (PROs) were collected from self-reported patient surveys at therapy initiation and while receiving treatment. Clinically significant cognitive impairment was defined as mild to severe symptoms measured on computer adaptive tests using the PROMIS Bank v2.0 — Cognitive Function. Multi-step analysis utilizing generalized estimating equations (GEE) was implemented to evaluate characteristics associated with cognitive function T-scores.

**Results:**

Our study included 51 ICI patients, with 51% being of Asian or Hispanic descent. Of 126 PROMIS symptom survey sets collected, 16.7% reported clinically significant cognitive impairment, with incidence peaking in survey sets collected 1–2 months removed from therapy initiation at 26.1%. All concurrent PROMIS symptom scores significantly correlated with cognitive function, including physical function (*r* = 0.33, *p* < 0.001), fatigue (*r* = − 0.61, *p* < 0.001), depression (*r* = − 0.56, *p* < 0.001), and anxiety (*r* = − 0.56, *p* < 0.001). Multi-variable regression demonstrated impaired physical function (Coef = − 4.01, *p* = 0.007), fatigue (Coef = − 5.15, *p* = 0.005), and anxiety (Coef = − 4.45, *p* < 0.001) are associated with decreased cognitive function scores, after adjusting for other patient characteristics.

**Conclusion:**

Patients receiving ICI therapy experience significant cognitive impairment with therapy initiation and in subsequent weeks and months during their therapy course. Managing and monitoring concurrent symptoms and inflammatory biomarkers may help identify at risk patients and alleviate cognitive impairments.

**Supplementary Information:**

The online version contains supplementary material available at 10.1007/s00520-025-09560-0.

## Introduction

Cognitive impairment experienced by cancer patients and survivors can be debilitating and lead to a significant decline in quality of life [1, 2]. Common presentations include an inability to concentrate, trouble multi-tasking, or memory lapses, which limit the ability of those affected to perform work, attend school, or engage in other social settings [3, 4]. The reported prevalence of cancer-related cognitive impairment (CRCI) varies widely within different patient populations, cancer types, and therapy exposures, but broad estimates suggest anywhere from 6 to 84% of cancer patients experience cognitive impairment [5, 6]. While chemotherapy has been consistently linked to CRCI symptoms, growing evidence posits that patients can experience CRCI at diagnosis before therapy or many years post-treatment as a survivor [7–9]. This suggests that the underlying causes of cognitive impairments are multifactorial.

One causal factor to CRCI may be dysregulated inflammatory pathways in cancer patients and survivors [10, 11]. Inflammation can be triggered by a variety of mechanisms in cancer survivors, including tissue damage from toxic therapies, immune response to cancerous tissue, and psycho-social stressors [11–14]. While the link between inflammation and neurocognitive decline continues to be explored, it is suggested that excessive circulating cytokines may disrupt and cross the blood–brain barrier, leading to neuro-inflammation and, thereby, neurotoxic effects [15]. Considering inflammation as a mediator of CRCI symptoms may explain why novel systemic anti-cancer therapies such as immunotherapies may also cause these symptoms.

Immune checkpoint inhibitors (ICI) are some of the most effective modern cancer therapies that block the inhibitory signals on T cells and thus improve therapeutic efficacy [16]. Undesired effects of ICIs are linked to dysregulated immune system activity, commonly known as immune-related adverse events (irAEs) [17]. These irAEs are mediated by inflammatory pathways, as excessive immune activity leads to undesired inflammation, which causes unwanted side effects [18, 19]. Multiple studies have demonstrated significant elevations of pro-inflammatory cytokines with ICI therapy and the occurrence of irAEs [20, 21]. Common presentations of irAEs include dermatitis, thyroiditis, and colitis. However, irAE-specific CNS toxicities also can occur [22, 23]. Given the role inflammation plays with CRCI and the associations of ICI therapy with elevated inflammation, cognitive toxicities within ICI patients merit further investigation.

Being recruited from a majority-minority county in California, our ICI patient population represents diverse ethnic backgrounds, socioeconomic statuses, and clinical presentations. While preliminary investigations have identified some risks with ICI therapy and cognitive toxicities, further validation in under-represented minority populations is needed. In this study, we utilize longitudinal observational data where ICI patients reported their symptom burden utilizing PROMIS-based patient-reported outcomes (PROs). Our study identifies the incidence of CRCI in ICI patients, provides unique insights into the risk factors for CRCI, and characterizes the cumulative symptomatic burden of patients on ICI therapy.

## Methods

### Study design

This was a prospective, longitudinal study that facilitated pharmacist-patient consultation visits to manage cancer and/or cancer treatment-related symptoms [24]. For each visit, participants reported their symptoms with NIH Patient-Reported Outcomes Measurement Information System (PROMIS)–based electronic surveys [25]. After trial initiation, the number of subsequent visits where symptom data was collected and their timing varied from patient to patient depending on their assessed needs, availability, and willingness to participate. Following each visit, participants would either be discharged from the study or scheduled for a follow-up visit based on mutual agreement between participants and clinical staff.

This study [24] was conducted within the University of California Irvine (UCI) Chao Family Comprehensive Cancer Center from July 2021 to June 2023. Study protocol received ethics approval from the UCI Institutional Review Board (#2021–6431), and all participants provided written informed consent before participation. Patients who did not wish to perform the research procedures or were physically and/or mentally incapable of providing written consent were excluded.

### Patient population

This study included adult patients (≥ 18 years) newly diagnosed with cancer and receiving ICI therapy, with PROMIS symptom surveys collected at therapy initiation with at least one additional measurement during ICI therapy (after initiation) for longitudinal evaluation. ICI therapies (monotherapy or in combination with chemotherapy) received included pembrolizumab, nivolumab, ipilimumab, atezolizumab, durvalumab, cemiplimab, or tremelimumab (Supplemental Table 1).

### Data collection

PROMIS was administered through REDCap using computer adaptive tests, which adapt the questionnaires based on patient responses to more reliably capture patient symptom severity and reduce unnecessary questions [26]. Patients were provided with an iPad before or during their infusion and completed assessments at their infusion chair. Measured health domains included cognitive function, physical function, anxiety, depression, and fatigue with patients completing PROMIS surveys for all domains. PROMIS symptom survey sets collected were categorized based on when they were collected relative to ICI therapy initiation (e.g., “within 1 month”, “within 2 months”, “within 3 months”). Surveys collected at the first infusion were labeled “therapy initiation” measurements.

Within each symptom domain represented in PROMIS surveys, the cumulative responses were totaled to generate a cumulative raw score. Following this, these scores were converted to normalized T-scores for ease of interpretation [27]. T-scores were transformed to level of severity (normal, mild, moderate, and severe impairment/symptoms) based on normative thresholds in real time ranging from 20 to 80 [26]. For functional domains of cognition and physical function, lower scores represent increasing impairment. We define T-scores below 45 as patients experiencing clinically meaningful impaired cognition or physical function based on the normative thresholds previously mentioned (mild, moderate, or severe impairment) [26]. For symptomatic domains of anxiety, depression, and fatigue, higher T-scores represent worsening symptoms. T-scores above 55 represent patients experiencing clinically meaningful symptoms based on previously mentioned normative thresholds (mild, moderate, or severe symptoms) [26].

The primary outcome of the study was based on self-reported cognitive function from collected PROMIS measures, utilizing both T-scores and incidence of clinically significant cognitive impairment (T-scores < 45 or mild, moderate, or severe symptoms) [27, 28]. Clinically significant concurrent symptoms (anxiety, depression, fatigue) or impairment (physical function) were similarly defined. Represented incidence rates of clinically significant symptoms and impairments are based on the proportion PROMIS surveys collected reflecting these impairments.

Other patient demographic information utilized in the study included age, race, ethnicity, marital status, employment status, and smoking history. Clinical information included body mass index (BMI), primary cancer type, and ICI therapy. To better account for measurement timing and therapy exposures, an exposure variable was created, labeling measurements as “initiation measurements,” “ICI monotherapy,” or “ICI with chemotherapy.”

### Statistical methods

#### Incidence of clinically significant symptoms

The incidence of self-reported CRCI was defined as the percentage of collected PROMIS symptom surveys reporting clinically significant cognitive impairment. The incidence of other clinically significant symptoms (anxiety, depression, fatigue) and impairments (physical function) identified with PROMIS data was similarly defined.

#### Cognitive function score trajectory over time

To highlight how cognitive symptom scores changed over time, linear trend plots were created. Mean cognitive function T-score was plotted over time, with time points represented as clusters of measurements within categorized time groupings (i.e., therapy initiation measurements, measurements between 1 and 2 months, between 2 and 3 months, etc.). These trends were also plotted and represented within distinct ethnic groupings to better understand observed toxicities within distinct patient populations.

#### Relationship between concurrent symptoms

Pearson correlation coefficients were calculated between the T-scores reported for each symptom domain for collected PROMIS surveys. The resulting coefficients and *p*-values are displayed in heatmaps, highlighting the significance and directionality of these relationships. Additionally, the incidence of clinically significant concurrent symptoms (anxiety, depression, fatigue, physical impairment) within PROs reporting cognitive impairments was compared to those reporting no impairment.

#### Statistical modeling

Multi-step analysis was conducted utilizing generalized estimating equations (GEE), adjusted for repeated measures with categorized measurement timing, and cognitive function T-scores were predicted as outcomes. Utilizing T-scores as an outcome (as opposed to clinically significant cognitive impairment) allowed for more robust modeling procedures with more predictors and multi-variable approaches. First, we conducted univariable analysis to identify which patient demographic, clinical characteristics, and concurrent clinically significant symptoms were associated with cognitive function scores. Features with *p*-values < 0.1 were selected and included in a multi-variable model predicting cognitive function T-scores. *P*-values considered for features within univariable and multi-variable models came from the Wald statistical test.

## Results

### Patient population

In total, 51 patients within the study received ICI therapy, with an average age of 62.7 years old (standard deviation [SD] = 14.3), and the majority of whom were male (70.6%), and married (72.5%) (Table 1; Supplemental Table 1). Roughly half the patient population was currently employed (51.0%) and had a history of smoking (47.1%) (Table 1). Non-Hispanic White patients were most represented (49.0%), followed by both Non-Hispanic Asian (23.5%) and Hispanic (27.5%) patients (Table 1). Of patients, 35.6% had normal (body mass index [BMI] < 25) BMIs, with the rest either being overweight (BMI ≥ 25) or obese (BMI ≥ 30) (Table 1). Melanoma (25.5%) was the most common primary cancer type, followed by genitourinary cancer (19.6% each), gastrointestinal cancers, and lung cancers (15.7% each) (Table 1). Most patients received ICI therapy alone (60.8%), with 39.2% receiving ICI therapy with chemotherapy (Table 1; Supplemental Table 1). Across primary malignancies within the study, there were notable differences in sociodemographic characteristics between cancer types (Supplemental Table 2). Mean age ranged by nearly 10 years between cancer sub-types, with the proportions of males and married patients varying by 20% between groups (Supplemental Table 2). Furthermore, distributions of different race/ethnicities, in additional education and employment status indicators, were highly variable across malignancies (Supplemental Table 2).

**Table 1:** Immune checkpoint inhibitor patient population characteristics. Categorical statistics are reported as percentages of the total patient population, with counts in parentheses. Mean and standard deviation (SD) are used to described numeric data

Category	Feature	Statistic (*n* = 51 total ICI patients)
Age	Age (SD)	62.7 (SD = 14.3)
Sex	Male patients	70.6% (*n* = 36)
Race/ethnicity	White (Non-Hispanic)	49% (*n* = 25)
	Asian (Non-Hispanic)	23.5% (*n* = 12)
	Hispanic/Latino	27.5% (*n* = 14)
Marital status	Married	72.5% (*n* = 37)
Employment status	Employed	51% (*n* = 26)
Education status	College graduate	49% (*n* = 25)
	High school graduate	23.5% (*n* = 12)
	Non-high school graduate	27.5% (*n* = 14)
BMI	Normal (BMI < 25)	33.3% (*n* = 17)
	Overweight (BMI ≥ 25)	43.1% (*n* = 22)
	Obese (BMI ≥ 30)	23.5% (*n* = 12)
Smoking status	History of smoking	47.1% (*n* = 24)
Primary cancer	Melanoma	25.5% (*n* = 13)
	Genitourinary cancer	19.6% (*n* = 10)
	Gastrointestinal cancer	15.7% (*n* = 8)
	Lung cancer	15.7% (*n* = 8)
	Other primary cancer	23.5% (*n* = 12)
ICI administration	ICI monotherapy	60.8% (*n* = 31)
	ICI with chemotherapy	39.2% (*n* = 20)

### Patient-reported outcomes collected

Amongst these patients, 126 PROMIS symptom survey sets were collected, with 51 occurring with ICI initiation and 75 occurring during ICI therapy (Supplemental Table 3). For measurements occurring during ICI therapy, 30 occurred during month 1 (1–30 days removed from therapy initiation), 23 during month 2 (31–60 days removed from therapy initiation), 11 during month 3 (61–90 days removed from therapy initiation), with 11 occurring more than 3 months removed from therapy initiation (Supplemental Table 3). Patient participation varied in the study, with 32 providing one survey set after initiation, 15 reporting two survey sets, and 4 reporting three or more (Supplemental Table 3). Collection timing of PROMIS symptom surveys widely varied from patient to patient relative to ICI initiation as well (Supplemental Table 4).

#### Incidence of clinically significant symptoms

Amongst all PROMIS symptom survey sets collected, the incidence of clinically significant cognitive impairment was 16.7% (Table 2). Incidence of other clinically significant symptoms included 50.8% physical impairment, 35.7% anxiety, 20.6% depression, and 30.2% fatigue. The peak incidence of cognitive impairment occurred between 1 and 2 months removed from therapy initiation, with 26% of surveys collected reporting impairment (Supplemental Table 5). For concurrent symptoms, the incidence of clinically significant symptoms also peaked for depression, fatigue, and physical impairment between 1 and 2 months removed from therapy initiation (Supplemental Table 5). Clinically significant anxiety peaked with therapy initiation measurements (Supplemental Table 5).

**Table 2:** Incidence of clinically significant symptoms within collected patient-reported outcomes. Statistics are reported as percentages of the total patient population, with counts in parentheses

**Symptom domain**	Statistic *n* = 126
Impaired cognition	16.7% (*n* = 21)
Impaired physical function	50.8% (*n* = 64)
Anxiety	35.7% (*n* = 45)
Depression	20.6% (*n* = 26)
Fatigue	30.2% (*n* = 38)

Amongst survey sets reporting clinically significant cognitive impairment, rates of clinically significant impaired physical function (76.2 vs. 45.7%), anxiety (76.2 vs. 27.6%), depression (57.1 vs. 13.3%), and fatigue (66.7 vs. 22.9%) were at least 20% higher compared to those without cognitive impairment (Table 3). These trends persisted when evaluated separately at therapy initiation and during therapy (Supplemental Table 6).

**Table 3:** Incidence of clinically significant symptoms amongst outcomes reporting cognitive impairments and not. From left to right, the first column represented the symptom domain captured in each row, followed by statistics amongst patients “not impaired” and then by “impaired” patients. Statistics are reported as percentages of the total patient population, with counts in parentheses

Symptom domain	Not impaired (*n* = 105)	Impaired (*n* = 21)
Impaired physical function	45.7% (*n* = 48)	76.2% (*n* = 16)
Anxiety	27.6% (*n* = 29)	76.2% (*n* = 16)
Depression	13.3% (*n* = 14)	57.1% (*n* = 12)
Fatigue	22.9% (*n* = 24)	66.7% (*n* = 14)

#### Cognitive function score trajectory over time

**Fig. 1:**
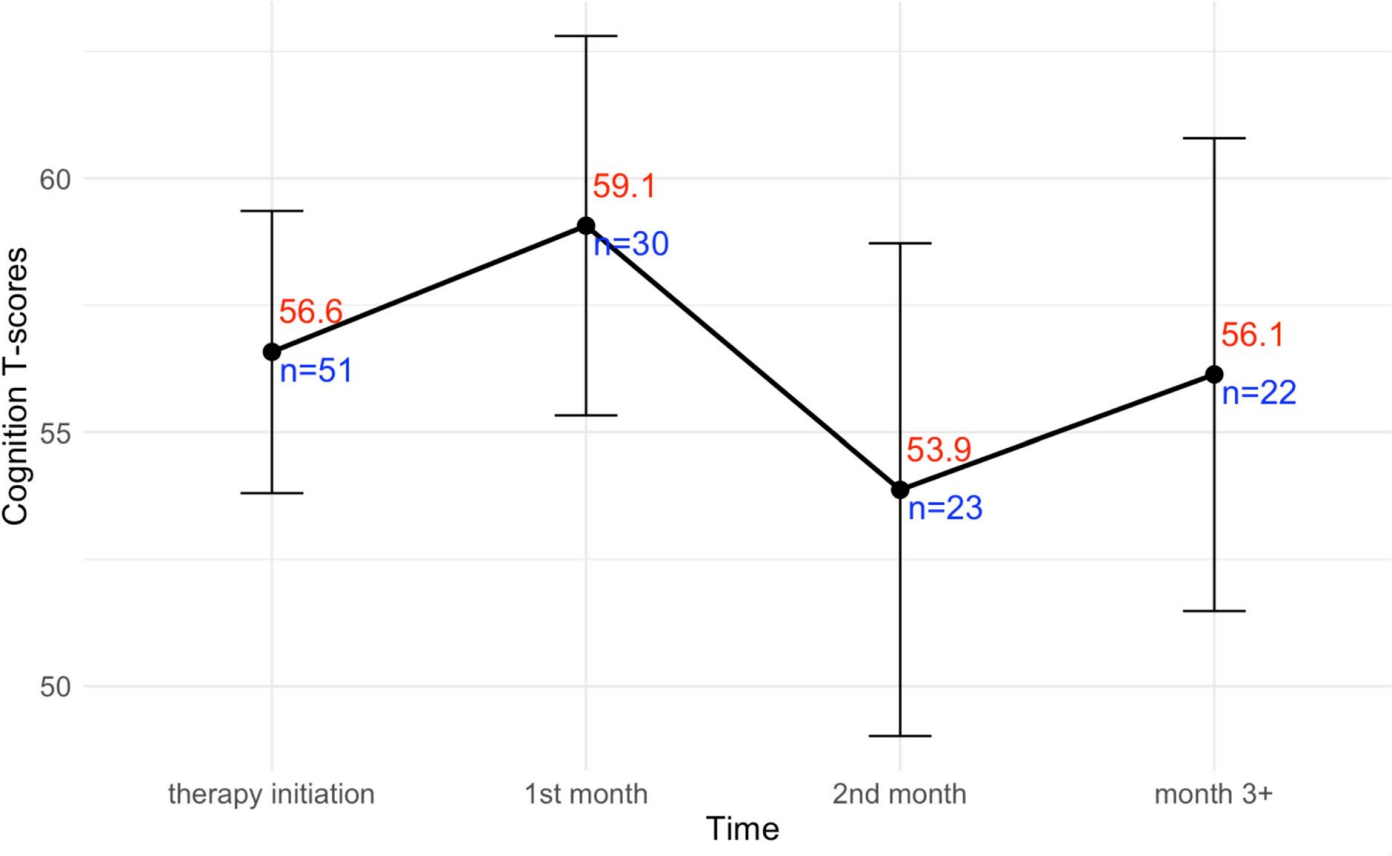
Trajectory of cognitive function scores over time. The *x*-axis represents categorized time groupings, while the *y*-axis represents cognitive function T-scores derived from PROMIS measurements. The points and numbers within the plot represent mean values, with the vertical lines and dashes representing error bars for 95% confidence intervals. Measurements occurring three or more months removed are grouped together in this plot due to limited sample size. Red numbers above each point indicate the mean cognitive function T-score of measurements within that time range, while blue number prefaced with *n* = indicate sample size at each time point, with all surveys collected during the study being represented in the plot

Amongst all patients, mean cognitive function T-scores are lowest within cognitive function measurements taken between 1 and 2 months removed from therapy initiation (Fig. 1). Mean values were three points lower relative to those taken at therapy initiation and six points lower relative to those taken within 1 month of therapy initiation. When considered within distinct racial/ethnic groups, mean values were lowest between 1 and 2 months removed from therapy initiation amongst White/Caucasian and Asian patients as well, while the symptom trajectory amongst Hispanics remained relatively constant (Supplemental Fig. 1).

#### Relationship between reported cognitive symptoms and other symptoms

**Fig. 2:**
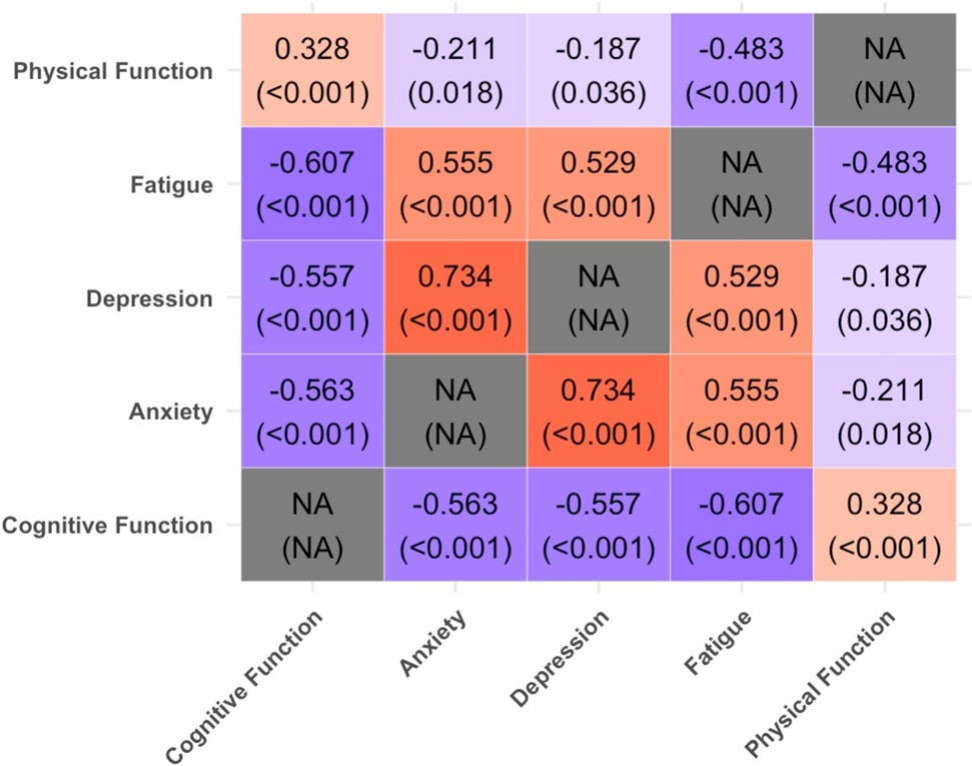
Pairwise Pearson correlation coefficients for PROMIS symptom T-scores. Each entry lists the correlation coefficient, with the corresponding p-value indicated below. Statistically significant positive correlations are highlighted in red, while significant negative correlations are shown in blue. Darker shades indicate stronger significance, whereas lighter shades suggest less significant associations

Amongst all PROMIS symptom T-scores collected, all other symptom domains have significant correlations with measured cognitive function scores, including physical function (*r* = 0.33, *p* < 0.001), fatigue (*r* = − 0.61, *p* < 0.001), depression (*r* = − 0.56, *p* < 0.001), and anxiety (*r* = − 0.56, *p* < 0.001) (Fig. 2). These significant associations persisted when considering measurements separately at therapy initiation and during therapy (Supplemental Fig. 2). Due to limited sample sizes, significant associations were not observed for PROMIS symptom T-scores when considered in categorized timing groups (i.e., measurements within 1 month, within 2 months, etc.), although the directionality of the relationships remained consistent (Supplemental Fig. 2).

#### Significant predictors of cognitive function T-scores

For univariable regression analysis, age at initiation (coef = − 0.21, *p* < 0.001), sex (female sex coef = − 2.9, *p* = 0.049, ref = male), marital status (not married coef = − 6.36, *p* < 0.001), race/ethnicity (Asian coef = 5.03, *p* < 0.001; Hispanic coef = 3.13, *p* = 0.022; ref = White), education status (high school graduate coef = 3.45, *p* = 0.048; no high school graduation coef = 5.73, *p* < 0.001; ref = college graduate), employment status (employed coef = 3.51, *p* = 0.035, ref = un-employed), lung cancer diagnosis (coef = 5.25, *p* = 0.001, binary variable), and genitourinary cancer diagnosis (coef = 4.01, *p* = 0.016, binary) (Supplemental Table 7). Clinically significant symptoms included physical impairment (coef = − 7.34, *p* < 0.001), anxiety (coef = − 9.55, *p* < 0.001), depression (coef = − 9.84, *p* < 0.001), and fatigue (coef = − 10.62, *p* < 0.001). Features representing BMI, therapy type, gastrointestinal cancer, and melanoma were not observed to have significant associations with cognitive function T-scores (Supplemental Table 7).

**Fig. 3:**
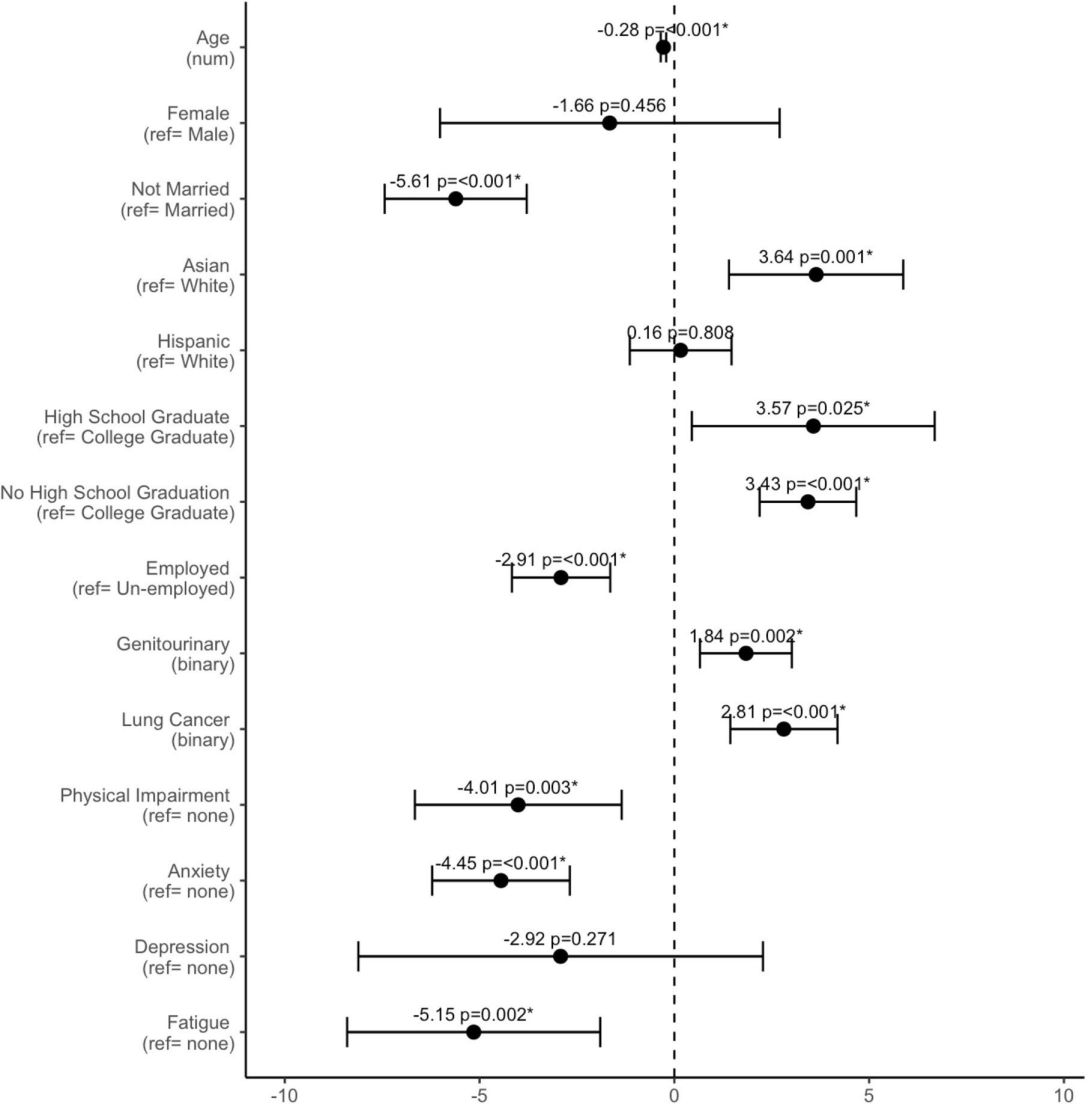
Multi-variable regression analysis predicting cognitive function T-scores. Each row represents a distinct predictive feature selected, with the *x*-axis representing reported coefficient values. Each point in the plot is the coefficient derived from regression analysis, with each associated line and dashed line representing its 95% confidence interval. Numeric data features are indicated as (num), with categorical features having their reference indicated (ref =). Features representing clinically significant symptoms are designated by the name of the symptom

In multivariable analysis, increasing age (coef = − 0.28, *p* < 0.001, numeric), marital status (not married coef = − 5.61, *p* < 0.001, ref = married), employment status (employed coef = − 2.91, *p* < 0.001, ref = un-employed), and clinically significant physical impairment (coef = − 4.01, *p* = 0.002, binary), anxiety (coef = − 4.45, *p* < 0.001, binary), and fatigue (coef = − 5.15, *p* = 0.002, binary) were all significantly associated with decreased cognitive function T-scores (Supplemental Table 8; Fig. 3). Additional significant factors within the multivariate model included Asian ethnicity (coef = 3.64, *p* = 0.001, ref = White), education status (high school graduate coef = 3.57, *p* = 0.025; non-high school graduate coef = 3.43, *p* < 0.001; ref = college graduate), along with genitourinary cancer diagnosis (coef = 1.84, *p* = 0.002, binary) and lung cancer diagnosis (coef = 2.81, < 0.001) which were associated with increased cognitive function T-scores. However, female sex, Hispanic ethnicity, and clinically significant depression were no longer significant predictors (Fig. 3; Supplemental Table 8).

## Discussion

Within our diverse study population, we identified the incidence of clinically significant CRCI utilizing PROMIS scores from self-reported surveys in a longitudinal observational study. While the observed cumulative incidence of impairment was comparable between measurements at therapy initiation and measurements during therapy (17.6 vs. 16.0%), there was an increase in cognitive impairment rate for surveys collected between 1 and 2 months removed from therapy initiation (26.1%). The incidence of clinically significant depression, fatigue, and impaired physical function also peaked during this time interval, a timing that is consistent with literature describing the average time to onset of various irAEs, including neurological ICI toxicities (~ 6 weeks) [29, 30]. The timing and peak incidence rates of impaired cognition in our study utilizing PROMIS measures are similar to larger studies evaluating CRCI in cancer survivors and other preliminary studies in ICI patient cohorts [5, 7, 31].

Studies have demonstrated that ICI exposures are associated with reduced measured cognitive function scores, although the immediate clinical implications of a reduced score can be challenging to interpret [32, 33]. In a recent study featuring a cohort of patients receiving ipilimumab, 23% experienced cognitive impairment as evaluated by subjective tests [34]. In another longitudinal study featuring a cohort of patients receiving pembrolizumab, 32% of patients experienced impaired cognition over time as assessed by objective computerized tests [35]. Our observed peak incidence of CRCI of around 26% appears to be consistent with current literature, suggesting that PROMIS measures can reliably capture clinically significant cognitive impairment in cancer patients.

We utilized a novel and growing approach to measure cognitive function in ICI-receiving patients using the PROMIS-Cognitive measure. Recent studies validated its associations with cancer-specific metrics, such as Functional Assessment of Cancer Therapy—Cognition (FACT-Cog) measurements [36]. This approach allows researchers to better contextualize cognitive impairment symptoms with concurrent measured PROMIS symptoms, as our patients demonstrated higher rates of clinically significant physical impairment, anxiety, depression, and fatigue with impaired cognition. Furthermore, we have utilized the computer adaptive tests, which help to decrease survey burden on our patients, a characteristic crucial for clinical implementation. Measured symptom scores in these domains were all significantly correlated as well, with worsening physical function, anxiety, depression, and fatigue strongly correlated with decreasing cognitive function scores. After adjustment with relevant confounders, clinically significant concurrent symptoms were associated with three-to-five-point reductions in measured cognitive function scores. Given this, increased efforts to screen for and treat these concurrent symptoms in ICI-treated patients may help improve or manage cognitive deficits [37].

Concurrent symptom burden as a risk factor for CRCI has been demonstrated in a variety of patient populations with respect to patient demographics, malignancy subtype, and therapy exposures [38–40]. Larger studies evaluating CRCI risk factors have shown associations with neuropsychiatric symptoms regardless of specific anti-cancer therapy exposures [41–44]. Physical function and fatigue symptoms also have associations with CRCI in different cancer cohorts, including patients recently completing chemotherapy and those many months removed from it [45, 46]. The link between ICIs and these symptoms themselves is also established, with several studies describing increased risk of a variety neuro-psych and fatigue with ICI administration [47–49]. Our results support the assertions of this body of literature describing the role of concurrent symptoms with CRCI, with cognitively impaired patients significantly more likely to have concurrent clinically significant symptoms and strong correlations observed between symptom scores. To better identify patients at risk for ICI-induced CRCI, increased efforts can be made to screen patients for concurrent symptoms throughout their treatment.

Our results demonstrated that race/ethnicity significantly influenced measured cognitive function, even when considered in context with other symptomatic and sociodemographic data. Asian patients had significantly higher measured cognitive function relative to white patients, even in context with confounders, and mean cognitive function measurements from Hispanic patients remained relatively constant over time, unlike those of White and Asian patients, which fluctuated. Recent CRCI studies have suggested significant disparities concerning its occurrence, showing that non-white individuals were consistently more likely to experience CRCI compared to white counterparts in several relevant studies [50]. With respect to broader ICI toxicities, there are several observed trends with respect to irAE incidence being influenced by Racial/Ethnic background [51]. Larger longitudinal prospective studies to investigate differences in ICI-related cognitive toxicities specific to different racial and ethnic populations are certainly warranted, taking additional steps to further validate potential disparities. While PROMIS symptom measurements have shown to be robust and are designed for use in diverse populations, the potential influence of different cultural norms on subjectively reported symptom data cannot be ignored [52]. The implementation of a neuropsychological test battery alongside subjectively captured measurements in future studies can further validate these toxicities and potential disparities with respect to race and ethnicity.

Our study highlighted many additional factors influencing measured cognitive function in ICI patients. Increasing age was associated with worsened cognitive function in our study, a finding consistent with existing literature [53, 54]. Consistent with our findings, previous studies have indicated that married patients are less likely to experience impairment, possibly due to increased support and care received. Employed patients had decreased cognitive function relative to unemployed patients within our cohort. There is growing literature describing the burden of cognitive toxicities on cancer patients in the workplace; perhaps stressful environments like the workplace bring a more acute awareness of impaired cognition [55]. Our results also reflect that patients of lower education status have higher measured cognitive function relative to those that have college degrees. Literature is mixed regarding the role of education status and self-reported cognitive function in cancer, with some suggesting more education increases awareness to report deficits, while others suggest an increased cognitive reserve makes them less likely to have meaningful decline [56]. While our observed associations with cognitive function measurements with sociodemographic factors are not unexpected, continued evaluations in large, diverse populations are warranted to further validate these associations.

With proliferating use of ICI agents due to their proven efficacy and broadening indications, the population of cancer patients using them become increasingly more diverse. While these agents were initially indicated for melanoma and lung cancers and often reserved for severe metastatic cases, this has changed in recent years [57]. Interestingly, our results show that certain primary cancer types influenced measured cognitive function measurements, with lung cancer and genitourinary cancer patients having increased cognitive function compared to other patients in the cohort. However, in a follow-up evaluation of our patient population, we found that the distribution of significant sociodemographic factors such as race/ethnicity and education status were significantly different across our patient population across cancer types. While cognition may vary based on primary cancer type, it is also possible that these findings could be attributed to sociodemographic differences or even other unobserved factors that could explain the phenomenon. Continued vigilant evaluation of ICI toxicities within this changing population is certainly needed, as distinct cancers have unique physiological properties that may predispose patients to cognitive toxicities [58].

Despite the incidence of clinically significant cognitive impairment in patients undergoing ICI therapy, the mechanisms of how ICIs induce these deficits are yet to be fully elucidated [56, 59]. CRCI may be linked to an elevated inflammatory response, where pro-inflammatory cytokines cross the blood–brain barrier and trigger neuroinflammation [60, 61]. ICIs have been demonstrated to be associated with circulating pro-inflammatory cytokines and increased T-cell activation, triggering downstream immune responses [19, 59, 62]. Microglial cells are a key player downstream in the immune response cascade that may play a role in ICI-induced CRCI mechanisms. In an emerging study (pre-print), ICI treatment was observed to trigger microglial activation, possibly leading to synaptic loss [59]. Furthermore, in addition to reduced pre- and post-synaptic protein densities, ICI treatment can also lead to loss of neuronal plasticity and myelin in vivo [59]. Other studies suggested that microglia may play a pivotal role, demonstrating that their activity and cytokine secretions increase with immune checkpoint blockade [63, 64]. Addressing cumulative immune dysregulation is challenging, given that traditional systemic immunosuppression may limit ICI efficacy. Pharmacologic agents promoting neuronal protection or formulated to suppress specific immune targets, including CNS immune cell pools and microglial activation, may be most beneficial.

While this study provides unique insights into ICI-related cognitive impairments, there were some limitations. Firstly, this pragmatic, real-world study involved a heterogeneous patient population concerning primary malignancy sub-type and anti-cancer therapy exposures; the incidence of CRCI may not be generalizable to other studies. The pragmatic nature of our study also leads to a mixed of number of reported outcomes per patient and variable reporting times relative to ICI initiation, making reliable population wide CRCI characterization not feasible. Our study also lacks supporting objective measures of cognition to support the identification of CRCI incidence in our patient population. While we attempted to account for different confounders of measured symptom scores, there were certain elements we could not reliably characterize (i.e., other prior medication exposures, comorbidities) which may contribute to measured cognitive function. Nevertheless, the study is one of the first to estimate and characterize cognitive impairment associated with ICI therapy, offering valuable insights into the survivorship care of patients receiving immunotherapies.

## Conclusion

Our study contributes to the growing body of evidence that ICI therapies have clinically meaningful cognitive toxicities. Our longitudinal study design, combined with the consideration of concurrent symptom burden with patient demographic information, provides unique insights into risk factors for CRCI within these patients. Utilizing PROMIS symptom tools to capture the symptomatic burden of cancer patients provided unique insights and allowed us to relate these symptoms more readily with one another. Its established standardized scoring practices and thresholds for clinically meaningful symptoms also allowed a more accessible translation of results into meaningful insights. Our study highlights that cognitive toxicities are relevant to a diverse racial/ethnic population of patients, and that more efforts need to be made to better understand risk factors specific to these unique populations. Larger longitudinal studies better defining the symptomatic burden of ICI patients utilizing these tools are needed, with healthy controls for comparison and measurements removed from active therapy to understand potential delayed toxicities.

## Supplementary Information

Below is the link to the electronic supplementary material.

ESM 1 (DOCX 493 KB)

## Data Availability

No datasets were generated or analysed during the current study.
