# Supplementary material for: Incidence and predictors of immune checkpoint inhibitor treatment–related cognitive impairment in a racial and ethnic diverse population
Source: Support Care Cancer. 2025 Jun 2;33(6):523. doi: 10.1007/s00520-025-09560-0 (PMC12127230; doi:10.1007/s00520-025-09560-0)
Supplement: Supplementary file 1 — (DOCX 493 KB) [file 520_2025_9560_MOESM1_ESM.docx]

**Supplemental Figures:**

**Supplemental Figure 1: Trajectory of Cognitive Function Scores Over Time Stratified by Race**

The x-axis represents categorized time groupings, while the y-axis represents cognitive function T-scores derived from PROMIS measurements. The points and numbers within the plot represent mean values, with the vertical lines and dashes representing error bars for 95% confidence intervals. Measurements occurring 3 or more months removed are grouped together in this plot due to limited sample size. Red numbers above each point indicate the mean cognitive function T-score of measurements within that time range, while blue number prefaced with n= indicate sample size at each timepoint, with all surveys collected during the study being represented in the plot

**
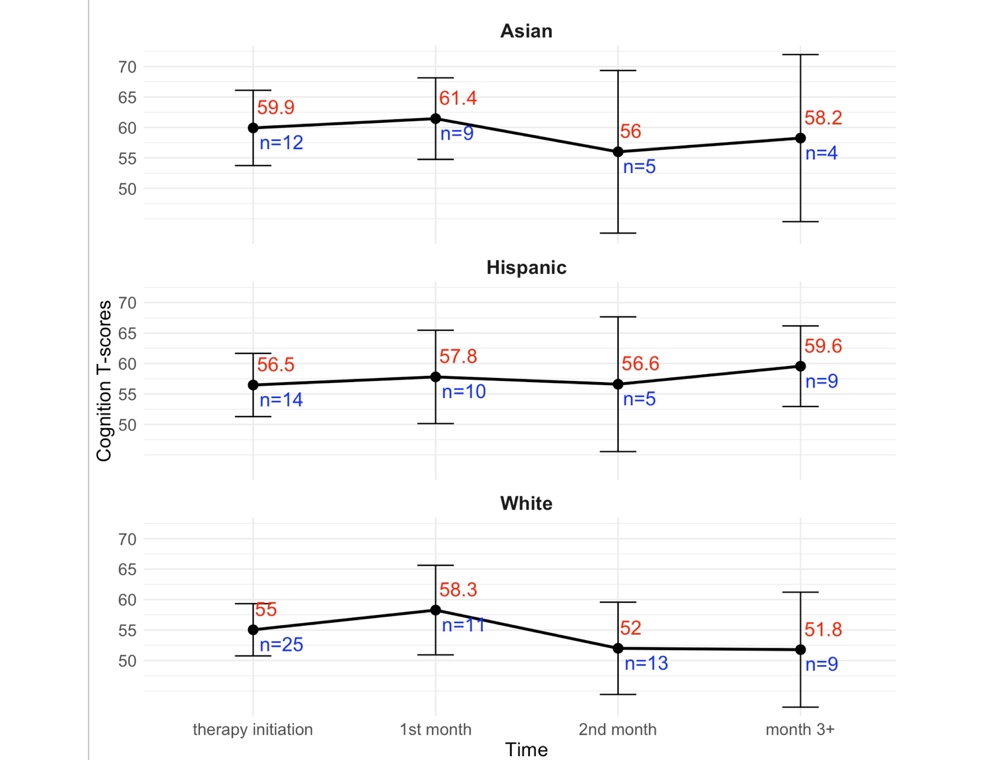
**

**Supplemental Figure 2: Pair-wise Pearson Correlation Coefficients for PROMIS Symptom T-Scores Within Categorized Sub-Populations Relative to Therapy Initiation**

Each entry lists the correlation coefficient, with the corresponding p-value indicated below. Statistically significant positive correlations are highlighted in red, while significant negative correlations are shown in blue. Darker shades indicate stronger significance, whereas lighter shades suggest less significant associations. Each plot represents categorized time frames, including therapy initiation measurements ("Therapy Initiation") and all results collected in visits after therapy initiation ("During Therapy"). Subsequent plots represent specific time frames for measurements collected after therapy initiation, including within 1 month (1-30 days), within 2 months (31-60 days), or 3 or months removed (60+ days). Measurements 3 or more months removed are grouped together due to limited sample size

**
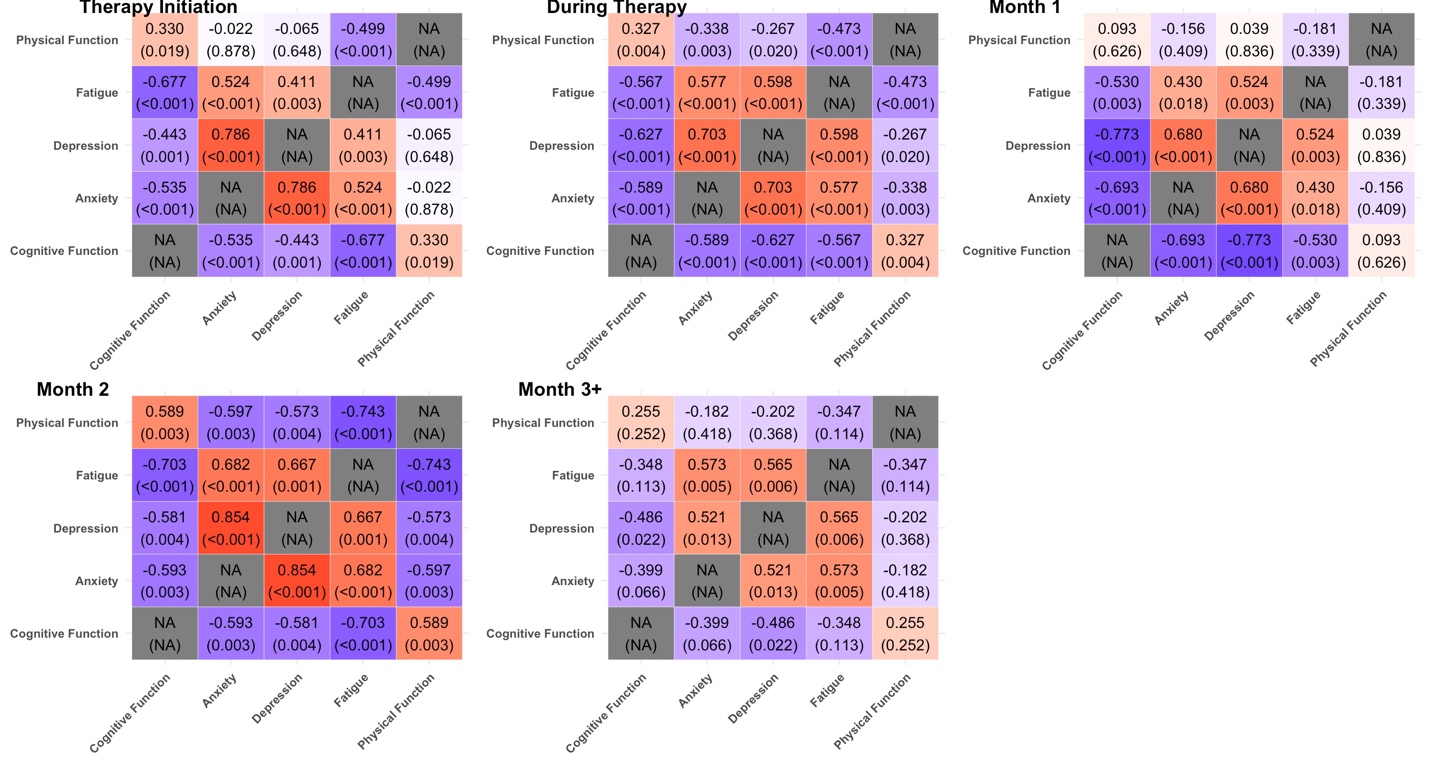
**

**Supplemental Tables:**

**Supplemental Table 1: Specific Immune Checkpoint Inhibitor Therapy Regimens Detailed**

Each row represents a distinct regimen implemented including immunotherapies and concurrent administration with chemotherapy. Percentages are reported with count data in parentheses, representative of the entire patient population

| **Regimen** | **Percent (Count)** |
| --- | --- |
| *atezolizumab* | 2.7% (n= 2) |
| *atezolizumab + chemotherapy** | 2.7% (n= 2) |
| *cemipilimab* | 2.7% (n= 2) |
| *durvalumab + chemotherapy* | 2.7% (n= 2) |
| *ipilimumab* | 1.4% (n= 1) |
| *nivolumab* | 4.1% (n= 3) |
| *nivolumab + chemotherapy* | 5.5% (n= 4) |
| *nivolumab + ipilimumab* | 16.4% (n= 12) |
| *nivolumab + ipilimumab + chemotherapy* | 1.4% (n= 1) |
| *pembrolizumab* | 39.7% (n= 29) |
| *pembrolizumab + chemotherapy* | 19.2% (n= 14) |
| *tremelimumab + durvalumab* | 1.4% (n= 1) |

**specific co-administered chemotherapies included: Carboplatin, Cisplatin, Docetaxel, Fluorouracil, Gemcitabine, Oxaplatin, Paclitaxel, and Pemetrexed*

**Supplemental Table 2: Patient-Reported Outcomes Collected at Therapy Initiation and During ICI Therapy**

Each column represents patients having a specific primary cancer sub-type, with the descriptive statistics for key sociodemographic factors detailed within each sub-cancer type population. Categorical statistics are reported as percentages of the specific patient population represented by the column, with associated counts in parenthesis

|  | **Melanoma** | **Genitourinary** | **GI** | **Lung** | **Other** |
| --- | --- | --- | --- | --- | --- |
| *Total Patients* | 13 | 10 | 8 | 8 | 12 |
| *Mean Age (Median)* | 58.6 (61) | 61.3 (62.5) | 59.9 (62) | 68 (68.5) | 66.8 (68) |
| *Male Sex* | 61.5% (n=8) | 90.0% (n=9) | 75.0% (n=6) | 87.5% (n=7) | 50.0% (n=6) |
| *Married* | 61.5% (n=8) | 90.0% (n=9) | 75.0% (n=6) | 75.0% (n=6) | 66.7% (n=8) |
| *White* | 84.6% (n=11) | 30.0% (n=3) | 37.5% (n=3) | 12.5% (n=1) | 58.3% (n=7) |
| *Hispanic* | 15.4% (n=2) | 40.0% (n=4) | 25.0% (n=2) | 25.0% (n=2) | 33.3% (n=4) |
| *Asian* | 0% (n=0) | 30.0% (n=3) | 37.5% (n=3) | 62.5% (n=5) | 8.3% (n=1) |
| *College* | 69.2% (n=9) | 50.0% (n=5) | 62.5% (n=5) | 12.5% (n=1) | 41.7% (n=5) |
| *Highschool* | 15.4% (n=2) | 20.0% (n=2) | 0% (n=0) | 50.0% (n=4) | 33.3% (n=4) |
| *No Highschool* | 15.4% (n=2) | 30.0% (n=3) | 37.5% (n=3) | 37.5% (n=3) | 25.0% (n=3) |
| *Employed* | 53.9% (n=7) | 60.0% (n=6) | 75.0% (n=6) | 50.0% (n=4) | 25.0% (n=3) |

**Supplemental Table 3: Patient-Reported Outcomes Collected at Therapy Initiation and During ICI Therapy**

Each row represents a distinct timing interval for sample and measurements collection, with the counts reported results within each window reported on the right.

|  | **Number of Measurements** |
| --- | --- |
| *Therapy Initiation* | 51 |
| *Total Measurements During Therapy* | 75 |
| *Month 1 (1-30 days)* | 30 |
| *Month 2 (31-60days)* | 23 |
| *Month 3 (61-90 days)* | 11 |
| *Month 4 (91-120 days)* | 1 |
| *Month 5 (121-150 days)* | 8 |
| *Month 6 (151-180 days)* | 1 |
| *Month 7 (181-210 days)* | 1 |

**Supplemental Table 4: Number of Distinct Measurements Collected From Patients and Their Timing Relative to Immune Checkpoint Inhibitor Therapy Initiation**

Survey number represents the numbered surveys collected characterized in chronological order relative to ICI initiation (each patients 1^st^ survey). Following this, the total number surveys collected representing each collection point (2^nd^ survey, 3^rd^ survey etc.), followed by the mean time removed from initiation that repeated collection represents, and lastly followed by the range of days represented for that collection.

| **Survey Number**  *(Relative to 1^st^ ICI Initiation Survey)* | **Total Measurements** | **Mean (Median) days Removed** | **Days Removed Range (Min- Max)** |
| --- | --- | --- | --- |
| **2nd** | 51 | 39.4 (28) | 7 - 144 |
| **3rd** | 19 | 81.5 (62) | 14 - 281 |
| **4th** | 4 | 91.8 (89) | 42 - 147 |
| **5th** | 1 | 129.0 (129) | 129- 129 |

**Supplemental Table 5: Incidence of Clinically Significant Symptoms with Collected Patient-Reported Outcomes.**

Each column represents a distinct timing interval measurements were collected, with incidence reported as a percentage, followed by the numeric count in parentheses.

|  | Therapy Initiation n= 51 | Post Therapy Initiation n= 75 | Month 1 n= 30 | Month 2 n= 23 | Month 3+ n=22 |
| --- | --- | --- | --- | --- | --- |
| **Impaired Cognition** | 17.6 (n=9) | 16 (n= 12) | 10 (n=3) | 26.1 (n=6) | 13.6 (n=3) |
| **Impaired Physical Function** | 47.1 (n=24) | 53.3 (n= 40) | 46.7 (n=14) | 60.9 (n=14) | 54.5 (n=12) |
| **Anxiety** | 43.1 (n=22) | 30.7 (n= 23) | 23.3 (n= 7) | 39.1 (n= 9) | 31.8 (n= 7) |
| **Depression** | 21.6 (n=11) | 20 (n= 15) | 10 (n= 3) | 30.4 (n= 7) | 22.7 (n= 5) |
| **Fatigue** | 27.5 (n=14) | 32 (n= 24) | 26.7 (n= 8) | 43.5 (n=10) | 27.3 (n= 6) |

**Supplemental Table 6: Incidence of Clinically Significant Symptoms Amongst Cases of Reported Cognitive Impairment at Therapy Initiation and During Therapy**

The first portion of the Table, labeled Concurrent Symptoms, presents the percentage of cases with clinically significant symptoms occurring with cognitive impairment. Values are reported separately for responses collected at therapy initiation and during ICI therapy.

|  | Therapy  Initiation | *n= 51* | During  Therapy | *n= 75* |
| --- | --- | --- | --- | --- |
| **Concurrent Symptom** | *Not Impaired* | *Impaired* | *Not Impaired* | *Impaired* |
| *Impaired Physical Function* | 42.9(n= 18) | 66.7(n= 6) | 47.6(n= 30) | 83.3(n= 10) |
| *Anxiety* | 33.3(n= 14) | 88.9(n= 8) | 23.8(n= 15) | 66.7(n= 8) |
| *Depression* | 16.7(n= 7) | 44.4(n= 4) | 11.1(n= 7) | 66.7(n= 8) |
| *Fatigue* | 16.7(n= 7) | 77.8(n= 7) | 27(n= 17) | 58.3(n= 7) |

**Supplemental Table 7: Univariable Regression Analysis Predicting Cognitive Function T-Scores**

Each row represents a distinct predictive feature considered. From left to right, each column represents the predictive feature names and their regression coefficient, followed by its 95% confidence interval and p-value. Numeric data features are indicated as (num), with categorical features having their reference indicated.

| **Predictor** | **Coefficient** | **95% Confidence Interval** | **P value** |
| --- | --- | --- | --- |
| *Age*  *(num)* | -0.21 | -0.32 to -0.11 | <0.001* |
| *Female*  *(ref= Male)* | -2.90 | -5.80 to -0.01 | 0.049* |
| *Not Married*  *(ref= Married)* | -6.36 | -9.39 to -3.32 | <0.001* |
| *Asian*  *(ref= White)* | 5.03 | 4.46 to 5.6 | <0.001* |
| *Hispanic*  *(ref= White)* | 3.13 | 0.46 to 5.8 | 0.022* |
| *High School Graduate*  *(ref= College Graduate)* | 3.45 | 0.03 to 6.87 | 0.048* |
| *No High School Graduation*  *(ref= College Graduate)* | 5.73 | 3.54 to 7.92 | <0.001* |
| *Employed*  *(ref= Un-employed)* | 3.51 | 0.25 to 6.77 | 0.035* |
| *BMI >= 30*  *(ref= BMI < 25)* | -0.88 | -4.07 to 2.32 | 0.591 |
| *BMI >= 25*  *(ref= BMI < 25)* | 0.07 | -2.34 to 2.49 | 0.952 |
| *History of Smoking*  *(ref= non-smoking)* | 0.03 | -3.36 to 3.42 | 0.985 |
| *Melanoma*  *(binary)* | -1.56 | -5.02 to 1.91 | 0.378 |
| *Genitourinary Cancer*  *(binary)* | 4.00 | 0.74 to 7.26 | 0.016* |
| *GI Cancer*  *(binary)* | 0.50 | -3.04 to 4.04 | 0.782 |
| *Lung Cancer*  *(binary)* | 5.25 | 2.18 to 8.32 | 0.001* |
| *ICI Monotherapy*  *(ref= Initiation Measurements)* | 0.27 | -1.60 to 2.13 | 0.780 |
| *Dual Therapy*  *(ref= Initiation Measurements)* | -0.22 | -4.06 to 3.63 | 0.911 |
| *Physical Impairment*  *(ref= none)* | -7.35 | -10.62 to -4.09 | <0.001* |
| *Anxiety*  *(ref= none)* | -9.55 | -11.66 to -7.44 | <0.001* |
| *Depression*  *(ref= none)* | -9.84 | -17.28 to -2.4 | 0.010* |
| *Fatigue*  *(ref= none)* | -10.62 | -12.38 to -8.85 | <0.001* |

**Supplemental Table 8: Multi-variable Regression Analysis Predicting Cognitive Function T-Scores**

Each row represents a distinct predictive selected for the multi-variable model. From left to right, each column represents the predictive feature names and their regression coefficient, followed by its 95% confidence interval and p-value. Numeric data features are indicated as (num), with categorical features having their reference indicated.

| **Predictor** | **Coefficient** | **95 % Confidence Interval** | **P-value** |
| --- | --- | --- | --- |
| *Age*  *(num)* | -0.28 | -0.35 to -0.21 | <0.001* |
| *Female*  *(ref= Male)* | -1.66 | -6.01 to 2.70 | 0.456 |
| *Not Married*  *(ref= Married)* | -5.61 | -7.44 to -3.79 | <0.001* |
| *Asian*  *(ref= White)* | 3.64 | 1.4 to 5.88 | 0.001 |
| *Hispanic*  *(ref= White)* | 0.16 | -1.14 to 1.47 | 0.808 |
| *High School Graduate*  *(ref= College Graduate)* | 3.57 | 0.45 to 6.68 | 0.025* |
| *No High School Graduation*  *(ref= College Graduate)* | 3.43 | 2.19 to 4.67 | <0.001* |
| *Employed*  *(ref= Un-employed)* | -2.91 | -4.17 to -1.65 | <0.001* |
| *Genitourinary*  *(binary)* | 1.84 | 0.66 to 3.02 | 0.002* |
| *Lung Cancer*  *(binary)* | 2.81 | 1.44 to 4.19 | <0.001* |
| *Physical Impairment*  *(ref= none)* | -4.01 | -6.66 to -1.35 | 0.003* |
| *Anxiety*  *(ref= none)* | -4.45 | -6.22 to -2.68 | <0.001* |
| *Depression*  *(ref= none)* | -2.92 | -8.11 to 2.27 | 0.271 |
| *Fatigue*  *(ref= none)* | -5.15 | -8.4 to -1.90 | 0.002* |
